# A framework for information technology-based management against COVID-19 in Iran

**DOI:** 10.1186/s12889-022-12781-1

**Published:** 2022-02-26

**Authors:** Afsoon Asadzadeh, Zeinab Mohammadzadeh, Zahra Fathifar, Soheila Jahangiri-Mirshekarlou, Peyman Rezaei-Hachesu

**Affiliations:** 1 Student Research Committee, Tabriz University of Medical Sciences, Tabriz, Iran; 2 Health Information Technology Department, School of Management and Medical Informatics, Tabriz University of Medical Sciences, Daneshgah St, 5165665811 Tabriz, Iran

**Keywords:** Information technology, COVID-19, Infectious disease, Outbreak, Epidemic

## Abstract

**Background:**

The COVID-19 pandemic has become a global concern. Iran is one of the countries affected most by the SARS-CoV-2 outbreak. As a result, the use of information technology (IT) has a variety of applications for pandemic management. The purpose of this study was to develop a conceptual framework for responding to the COVID-19 pandemic via IT management, based on extensive literature review and expert knowledge.

**Methods:**

The conceptual framework is developed in three stages: (1) a literature review to gather practical experience with IT applications for managing the COVID-19 pandemic, (2) a study of Iranian documents and papers that present Iran’s practical experience with COVID-19, and (3) developing a conceptual framework based on the previous steps and validating it through a Delphi approach in two rounds, and by 13 experts.

**Results:**

The proposed conceptual framework demonstrates that during pandemics, 22 different types of technologies were used for various purposes, including virtual education, early warning, rapid screening and diagnosis of infected individuals, and data management. These objectives were classified into six categories, with the following applications highlighted: (1) Prevention (M-health, Internet search queries, telehealth, robotics, Internet of things (IoT), Artificial Intelligence (AI), big data, Virtual Reality (VR), social media); (2) Diagnosis (M-health, drones, telehealth, IoT, Robotics, AI, Decision Support System (DSS), Electronic Health Record (EHR)); (3) Treatment (Telehealth, M-health, AI, Robotic, VR, IoT); (4) Follow-up (Telehealth, M-health, VR), (5) Management & planning (Geographic information system, M-health, IoT, blockchain), and (6) Protection (IoT, AI, Robotic and automatic vehicles, Augmented Reality (AR)). In Iran, the use of IT for prevention has been emphasized through M-health, internet search queries, social media, video conferencing, management and planning objectives using databases, health information systems, dashboards, surveillance systems, and vaccine coverage.

**Conclusions:**

IT capabilities were critical during the COVID-19 outbreak. Practical experience demonstrates that various aspects of information technologies were overlooked. To combat this pandemic, the government and decision-makers of this country should consider strategic planning that incorporates successful experiences against COVID-19 and the most advanced IT capabilities.

## Background

Recently, coronavirus 2019 (COVID-19) emerged as a global infection disease caused by severe acute respiratory syndrome coronavirus 2 (SARS-CoV-2) in Wuhan, China, on December 31, 2019, has since spread worldwide [1]. The primary concern in the COVID-19 outbreak is the pathogen’s ability to infect humans and cause a variety of clinical symptoms and severity [2]. Patients with COVID-19 frequently experience fever, dry cough, muscle ache, fatigue, shortness of breath, weakness, chest tightness, nasal congestion, and sore throat [3]. This pandemic has significantly impacted numerous facets of human life, including public health, well-being, businesses, education, and entertainment [1, 4].

Presently, SARS-CoV-2 has been detected in 222 countries [5], with Iran being one of the countries most affected by the virus [6]. The first COVID-19 cases in Iran were reported on February 19, 2020, and by March 5, 2020, all 31 provinces of Iran had been infected [7]. By July 5, 2021, the number of confirmed patients in this country was 3,270,843, with 84,949 deaths and 2,940,874 recoveries reported [5]. Following a sharp increase in COVID-19 cases, governments worldwide are implementing plans, including restriction and management strategies, to contain and prevent the current pandemic’s spread [8]. IT is viewed as a critical component of emergency management for the COVID-19 pandemic in various dimensions, including mitigation, preparedness, prevention, diagnosis, screening, treatment, and recovery [9, 10] IT is defined as the use of computers and other information technology to acquire, organize, store, retrieve, and disseminate information [11]. IT can significantly reduce the costs associated with coordination, communication, and information processing [12].

During this pandemic, several studies have been conducted to demonstrate IT applications’ effectiveness against COVID-19 [9, 13–15]. There is a need to conduct studies to illustrate each country’s practical experience with IT-based COVID-19 management to determine which digital solutions should be emphasized or ignored [9]. It should be noted that presenting the conceptual framework for managing current pandemics based on IT potentials will help inform government, policymakers, software designers, informatics, and health information technology experts on how to use and plan IT-based solutions for the COVID-19 epidemic’s emergency management. As a result, the primary goal of the current study was to develop a framework for managing pandemics based on IT’s practical experience in Iran. Then, based on extensive literature and expert knowledge, a comprehensive framework for managing the COVID-19 pandemic is proposed.

## Methods

**Fig. 1:**
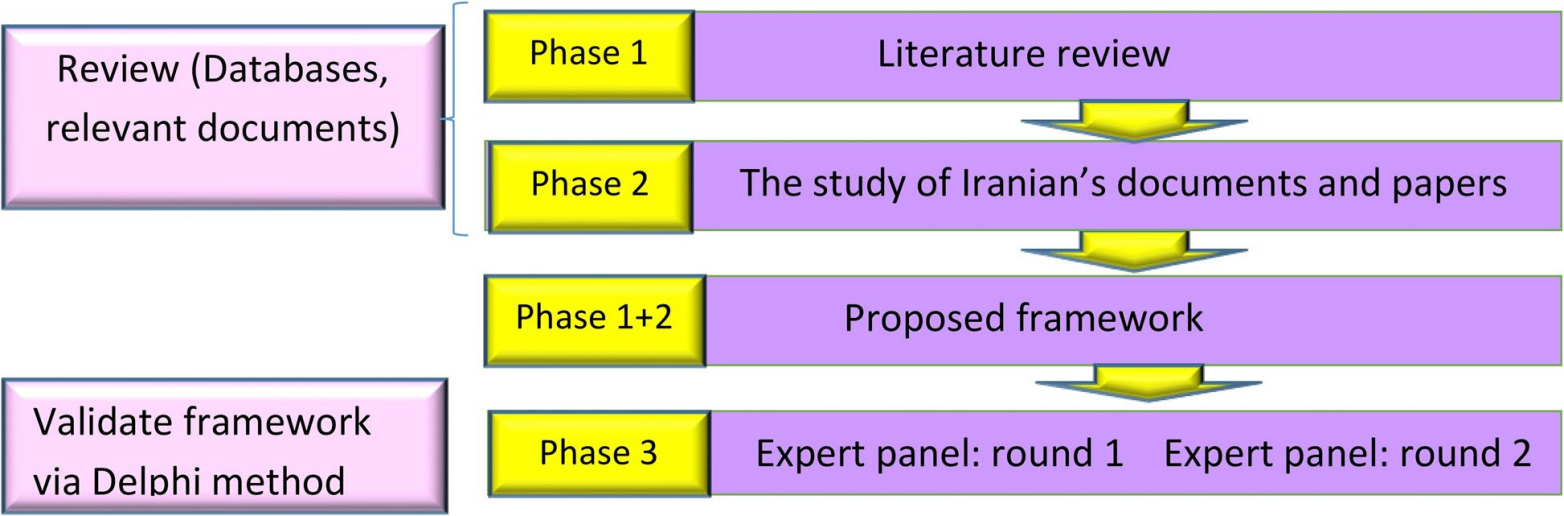
An overview of the methods’ steps

There are three phases to the current study’s methodology (Fig. 1). The papers identified from the information sources (see Section Databases and search strategy) were screened according to inclusion and exclusion criteria (see Section Inclusion and Exclusion criteria) by three authors (AA, PRH, and ZM). They reviewed the full text of included papers demonstrating various IT solutions used during the COVID-19 pandemic. The information of forty articles were extracted and shown in Table 2. Each gathered IT solution was searched through Iranian databases and official websites during the second phase to determine which technologies were used. The third phase proposed an initial framework based on the findings of the first and second phases, which were then sent to experts for validation and completion. The following figure depicts an overview of the three phases.

### Phase 1. Literature review

#### Databases and search strategy

A literature review was conducted to identify studies that documented the use of IT in the management of the COVID-19 outbreak. We selected appropriate keywords based on related articles and the advice of experts in health information technology, health information management, and medical informatics (Table 1). MEDLINE (PubMed), Embase, IEEE, Scopus, Web of Science, Google Scholar, and the Google search engine were used to search keywords between December 2019 and April 2021. The current study’s search strategy is summarized in Table 1.

**Table 1:** The search strategy

Search	Details
#1	**[**“covid 19“[Title/Abstract] OR “2019 ncov“[Title/Abstract] OR “2019 cov“[Title/Abstract] OR “coronavirus“[Title/Abstract] OR “novel coronavirus“[Title/Abstract] OR “coronavirus 2019“[Title/Abstract] OR “sars cov 2“[Title/Abstract] OR “SARS-CoV-2” OR “coronavirus disease 2019“[Title/Abstract] OR “Severe acute respiratory syndrome coronavirus 2“[Title/Abstract] OR “COVID-19“[Mesh]**]**.
#2	“Information Technology“[Mesh] AND “information technology” [Title/Abstract] OR “IT” [Title/Abstract] OR “Informatics” [Title/Abstract] OR “informatics” [Title/Abstract] OR “computer technology” [Title/Abstract] OR “electronic technology” [Title/Abstract] OR “Information and Communication Technology” [Title/Abstract] OR “ICT” [Title/Abstract] OR “computerized information” [Title/Abstract] OR “Information system” [Title/Abstract] OR “Infotech” [Title/Abstract] OR “data processing” [Title/Abstract]
#3	#1 AND #2
	Limits: English language

#### Inclusion and exclusion criteria

This study included review articles on IT solutions, peer-reviewed studies, and English language papers. Letters to the Editor, opinions, protocols, and studies unrelated to the topic were excluded.

#### Data extraction

The identified publications were added to an EndNote X8 library, and duplicates were eliminated. Three authors screened the articles in three stages: (1) Title, (2) Abstract and (3) Full text. Disagreements with the fourth and fifth authors were resolved via an online meeting. The following data were extracted for the COVID-19 pandemic: authors, IT types, and applications of the technologies included. Finally, the extracted applications were used to classify the results into sub-themes.

### Phase 2. IT used for COVID-19 in Iran

Related papers or documents for IT applications in Iran were extracted from (1) phase 1 (see Section Phase 1. Literature review), (2) official Iranian websites (i.e., the Ministry of Health and Medical Education, academic, and “The National Headquarters for Coronavirus Control”), and (3) Iranian databases including SID, IRAN MEDEX, and Islamic World Science Citation Center (ISC). Published documents between December 2019 and April 2021 were reviewed by all five authors.Finally, a conceptual framework for IT-based management of the COVID-19 was provided based on this country’s practical experiences.

### Phase 3. Proposed conceptual framework and validation

Based on the results from the first and second phases, a comprehensive conceptual framework was developed. It was distributed to experts with Ph.D. degrees and relevant experience, including health information technology/health information management (*N *= 11) and information technology (IT) experts (*N *= 3), who were asked to validate and apply their knowledge within the proposed framework for completion during 20 days. The framework was modified in response to expert recommendations, and the framework’s vulnerabilities were addressed. Finally, they were contacted to finalize the conceptual framework.

## Results

### Phase 1: Literature review for IT-based management of COVID-19

Phase 1 included forty review articles. Based on the results, the major components of IT used for COVID-19 have been classified and summarized in Table 2.

**Table 2:** IT applications in the COVID-19 pandemic

Objectives	IT types (References)	Applications
Prevention	Mobile health(M-health) [4, 9, 16–28]	* Virtual education and training * Information sharing * Remote consultation * Providing updated information on newly suspected COVID-19 communities/regions * Raising awareness * Location monitoring * Monitoring the movements of quarantined individuals * Promoting personal health tracking * Crowd activity monitoring * Contact tracing (initial screening, have a “chatbox” for real-time communication, education, and remote monitoring) * Promoting personal health tracking
	Internet search query [9, 13, 17, 29]	* Raising public awareness and social control * Online tracking system for the COVID-19 pandemic * Internet search data demonstrates information-seeking behavior in response to local COVID-19 cases and web-search behavior. * Monitor the disease outbreak and information dissemination * Health Education and Promotion * Internet-based consultation sessions
	Robotic [9, 13, 16, 23, 29, 30]	* Allows doctors to monitor and interact with infected patients * To assist doctors in taking vital signs and communicating with patients via a large screen in Seattle * To transport medical supplies * Navigates and sanitizes floors without human interference * Spraying disinfectants * Cleaning
	Telehealth [9, 13, 17, 20, 22, 29–33]	* Consulting (e.g., Synchronous video consultation) * Health Education and Promotion * Virtual education * A self-assessment conducted asynchronously through an application to determine possible symptoms * Remote patient monitoring
	Internet of Things (IoT) [9, 23, 29, 30, 34–36]	* Monitor and measure the level of container * Data collection/sharing * For social distancing * Remote monitoring of COVID-19 patients in quarantine centers and/or self-isolation * Contact Tracing and Clustering
	Artificial Intelligence (AI) [16, 23, 29, 30, 35, 37–43]	* Provide updated information * Early warnings and alerts * Awareness and social control * Monitoring and maintaining social distance * Tracking pedestrians
	Big data analytics [4, 29, 30, 35, 42, 44]	* Raise awareness * Raise education
	Virtual Reality (VR) [9, 29, 30, 45]	* Educating and learning about the COVID-19 virus * Tele-communication to share patient experiences * Educating children on coronavirus safety
	Social media [13, 17, 21, 29]	* Early warning * Health Education and Promotion * Information dissemination
	Blockchain [23, 46]	* Preventing the circulation of fake news * To share COVID-19 data on the Algorand blockchain
	Video games [13]	* Education of protection strategies
	Drones [13, 29]	* Spraying disinfectants in remote areas * To deliver medical supplies
	5G network [23]	* Facilitates teleconsultation * Providing better assistance to the frontline staff and facilitating improved virus tracking
	Decision support system (DSS) [17]	* Early warning
	Surveillance system [9]	* To identify quarantined individuals with COVID-19 symptoms
	Augmented Reality (AR)[45]	* Providing high-resolution audio and video
	Cloud computing [17]	* Early warning
Diagnosis	M-health [9, 16–22, 24, 27, 28, 31, 47]	* Telemedicine visitation services and virtual venues for meetings * Virtual education * Training * Information sharing * Remote consultation * Raise awareness * Location monitoring * Clinical Symptom Monitoring * Unobtrusive Sensing for Physiological and Symptomatic Monitoring of COVID-19 * Multi-parameter Physiological Monitoring
	Drones [29]	* Allows to scan through a crowd of people and detect someone in need of medical attention (crowd surveillance) * To monitor citizens in real-time * Delivery of medical supplies and other essentials (e.g., thermal imaging camera) and patient samples to improve virus detection * Public announcements
	Telehealth [9, 17, 20, 22, 30, 31, 33]	* Assessing patients (assess the possibility of being a COVID-19 carrier) * To support nurses and doctors in screening sick patients * To rapidly screen potential COVID-19 cases using questionnaires * Remotely monitoring and diagnosing * Providing health services using virtual care for clinical examinations
	AI [23, 29, 30, 37–40, 42, 43, 48–50]	* Diagnosis of COVID-19 from Chest CT Images * Diagnosis using radiology images * Screening the patient risk * Early detection * CT assessment of patients * The severity of COVID-19 * Face Mask Detection * Thermography * Germ screening * Risk Assessment and Patient Prioritization
	IoT [4, 9, 23, 30, 34, 36]	* Real-time Tracking (e.g., To track clinical specimens) * Rapid and enhanced diagnosis * Screening * Smart thermometers to screen people for high fevers
	DSS [9]	* Early detection * Screening and triage * Risk assessment for suspected individuals * To assist general practitioners (GPs) * For characterizing the severity of COVID-19
	Robots [9, 16, 29]	* Measuring body temperature and recording other relevant data * Assisting patient diagnosis
	(Geographic information system) GIS [9, 17]	* COVID-19 contact tracing * Track COVID-19 patients * Triage of COVD19 patients
	5G-network [23]	* Facilitating improved virus tracking * Facilitates teleconsultation
	Big data analytics [42, 44]	* Assist with diagnosis * Estimate or predict risk score
	Surveillance system [9]	* Early detection
	VR [45]	* Seeing into the patient’s lungs
	Blockchain [23]	* Facilitating increased testing
	Video games [13]	* Assist with diagnosis
	Social media [46]	* Assist with early detection
	(Electronic health record) EHR	* Assist with quick diagnosis
	3D printing [46]	* Meeting the requirements of medical equipment
	Nanotechnology [46]	* Can help to diagnose and treat through Nanoparticles and equipment
	Cloud computing [17]	* Via mobile system: Early detection, screening, and triage
Treatment	Telehealth [9, 13, 20, 22, 30–33]	* Monitor patient recovery * Outpatient treatment * Communication between patients and physicians through secure video chats * Remote patient monitoring * Virtual care * Chronic disease management * Treatment of sleep disorders * Elderly care
	M-health [9, 16–24, 27, 31]	* Telemedicine visit services and virtual venues for meetings * To monitor patients with mild symptoms who have tested positive for COVID-19 * Self-management of symptoms * Remote consultation * Monitor patients (by temperature, heart rate, oxygen, and blood pressure) * Improve mental health (Remote Physiological Monitoring) * Monitor people’s physical health, along with their stress levels
	AI [23, 29, 30, 37–40, 42]	* Identification of a potential therapeutic option * Critical patient screening * Support vaccination development * Automated Patient Care * Monitoring * Development of drugs and vaccines
	Robotic and automatic vehicles [9, 16, 23, 29]	* Monitoring the quarantined patients * Monitor patient recovery * Allows doctors to monitor and interact with infected patients * To assist in the treatment of patients * Advice on symptomatic management and self-isolation
	VR [45]	* In palliative care * Telehealth VR system for various disorders * Assisting in the discovery of potential molecular targets for the inhibition of COVID-19 proteins
	IoT [4, 9, 23, 30, 34, 36]	* Remote patient monitoring * Effective monitoring and control * To facilitate remote patient monitoring
	Big data analytics [44]	* Healthcare decision making * Pharmaceutical
	5G network [23]	* Patient monitoring * Facilitates Teleconsultation
	Video games [13]	* Help drug discovery: helped immensely in reconstructing the structure of the coronavirus protein, helps in (virus) protein three-dimensional (3D) reconstruction and similarity searching
	Bioinformatics systems [9]	* Drug discovery
Protection	IoT [4, 9, 23, 34–36]	* Control hazardous waste * Real-Time Waste Inf. * Remote monitoring * IoT BUTTONS To maintain high cleaning standards and limit the number of hospital-acquired infection * IoT solutions for social distancing
	AI [23, 29, 35, 37–40, 42]	* Reducing the workload of healthcare workers * Facilitating the implementation of public health interventions * Control hazardous waste
	Robotic and automatic vehicles [9, 13, 16, 23, 29]	* Reducing the workload of healthcare workers * Facilitating the implementation of public health interventions * Spraying disinfectants
	AR [45]	* Sending patient information to the healthcare system directly * Visualization of invisible concepts
	Surveillance system [9]	* Enhancing staff protection in a healthcare setting
	Telehealth [9, 20, 22, 31, 33]	* The state pharmacy boards have temporarily permitted pharmacists to remotely work and carry out dispensing activities outside a licensed pharmacy * Reducing face to face visits
	M-health [17, 18, 24, 25, 27, 28]	* Self-management of symptoms * Mobile telehealth system * People and health care providers have benefited from mobile phone diagnosis, treatment, monitoring, and screening in any location, which has resulted in a reduction in touch.
	Big data analytics [35]	* Control hazardous waste
	Drones [23]	* Minimize human interaction
Management aims	GIS [4, 9, 17, 51, 52]	* COVID-19 Data Visualization and Exploratory Data Analysis (i.e., dashboards * Spatiotemporal analysis * Health and social geography * Web-based mapping * Provide information related to the surveillance of the pandemic * Real-time tracking of transmission, activity tracking, and quarantine-level analysis * Real-time tracking map * Spatial modeling * Provide data mining data
	M-health [9, 17–20, 23, 24, 27, 28]	* Information sharing * Assist in planning further management * Current status and statistics from local areas (specific countries) to a global overview * Providing updated information on newly suspected COVID-19 communities/regions * Monitor the disease outbreak and information dissemination * Resource allocation * Visualization of the State governments’ data * Crowd activity monitoring * Scatter contact person mobility patterns
	Blockchain [4, 23, 30, 35, 46]	* Facilitating increased reporting * Recording COVID-19 patient data * Managing lockdown implementation * Enabling an incentive-based volunteer participation platform * Enabling a secure donation platform for sponsors * Limiting supply chain disruptions * Support for immunity and vaccine certification * Route setting *Authenticate and validate COVID-19 and encrypt health data
	IoT [4, 9, 30, 34–36]	* Surveillance * To control the amount of hazardous waste and monitor the amount of waste * Data collection/sharing * Ability to transfer data over a computer network * Real-time access to COVID-19 data
	AI [23, 29, 30, 35, 37–40, 42]	* Prediction of a patient’s health condition’s outcome * Protein structure predictions * Predict the severity of COVID-19 * The spread and progression of the pneumonia lesions * Predicting affected patients * Risk Prediction * Debunking fake news * Enforcing lockdown measures * Social control * Control hazardous waste
	5G network [23]	* Data collection and analysis * Perceived quality of service and data transfer rate to ensure real-time access to data
	internet search queries [9]	* Showing information flow of the COVID-19 pandemic * Epidemiologic surveillance tool
	Telehealth [9, 20]	* Resource allocation * Outbreak surveillance
	Big data analytics [4, 29, 30, 35, 44]	* Collecting, sorting, and analyzing the huge masses of data * Connected to national health information systems (HIS) and store data (mobility patterns) on a real-time basis for further analysis * Healthcare decision-making * Control hazardous waste
	DSS [9, 35]	* Use in the healthcare supply chain * Help to demand management in the health care context
	Social Media [17]	* The dynamic burden of the pandemic and analysis of its consequences
	Surveillance system [9]	* To estimate COVID-19 growth rate through surveillance systems * Timely updates of references and population’s data
	Video games [13]	* Assists with the transformation of large amounts of data into visual images
	EHR [53]	* Assist with health data analysis * Assist with prediction by providing health data
	Cloud computing [35]	* Through big data analytics: Control hazardous waste

As a result of our findings, the IT objectives for COVID-19 can be classified into six categories: (1) prevention and control (i.e., actions taken during the pandemic to prevent further outbreaks), (2) diagnosis and detection, (3) treatment, (4) follow-up, (5) protection strategies (i.e., the safety of health care providers and the general public during the pandemic), and (6) management and planning strategies. Table 2 details these classifications and their associated cases.

### Phase 2. The examination of Iranian documents and papers

**Fig. 2:**
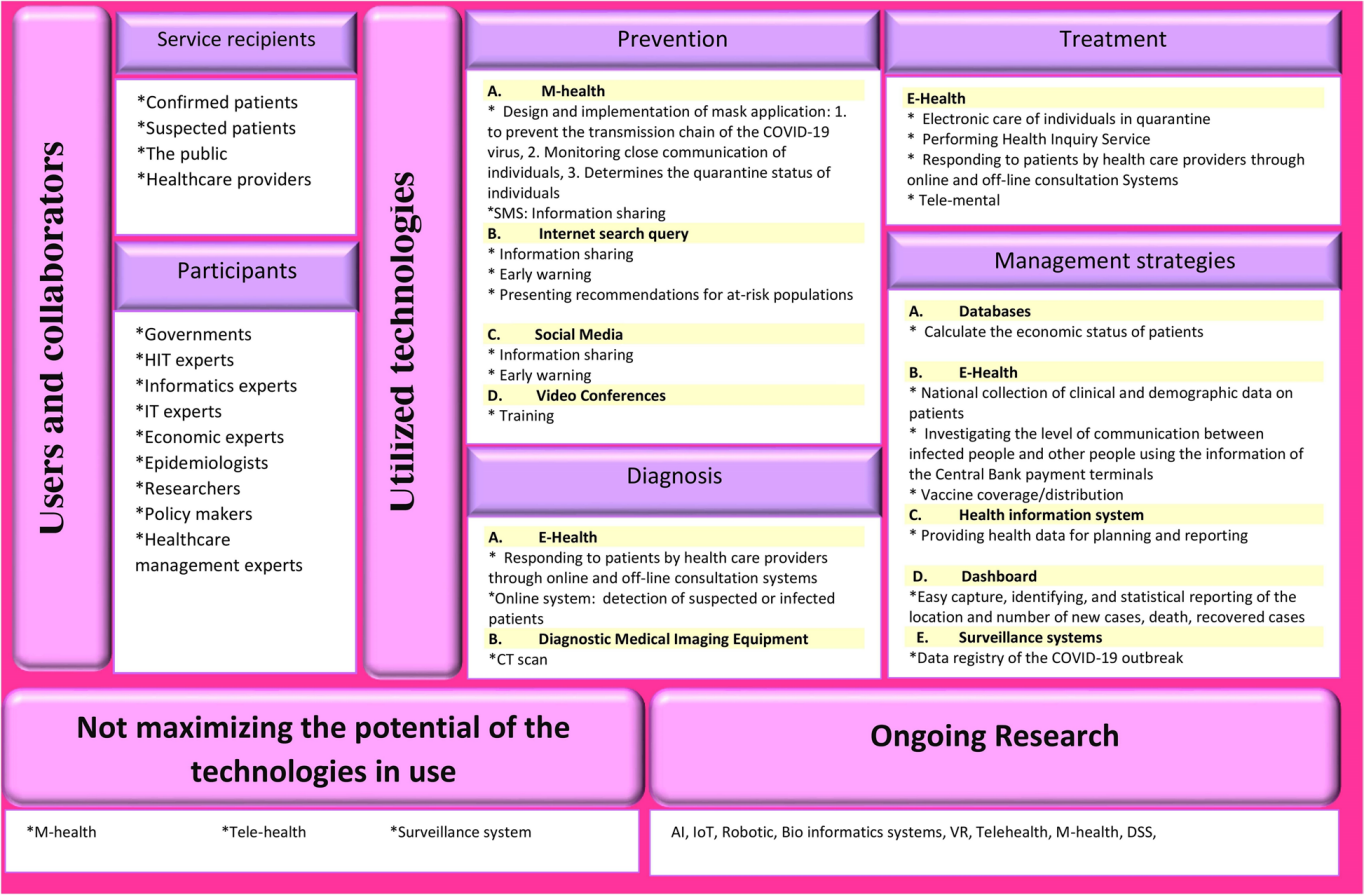
A conceptual framework for managing the COVID-19 pandemic in Iran based on practical IT experience

Iran’s approach to managing COVID-19 through IT capabilities are depicted in Fig. 2. The information was completed and validated with the assistance of experts.

In Iran, specific IT applications have been used to accomplish four primary goals: (a) Prevention, (b) Diagnosis, (c) Treatment and (d) Management and planning (see Fig. 2 for details).

- *Prevention*

The “Mask” application is a mobile application developed in Iran to educate the public about COVID-19. This app displays the most recent news regarding COVID-19 and the infection rate in Iran’s cities. Additionally, this app’s purpose is to aid in the initial identification of infected or suspected cases by asking questions about an individual’s physical condition [54]. WhatsApp, Telegram, Instagram, Rubika, Facebook, and Twitter have all been extensively used to share information and personal experiences about the pandemic [55]. Video conferences are another method of sharing knowledge about prevention strategies [13].

Furthermore, official websites such as those of the Ministry of Health and Medical Education and academic websites are used to educate the public about preventative measures [56, 57]. The Executive Headquarters of Imam Khomeini developed the “4030” call service in collaboration with the Ministry of Health, Treatment, and Medical Education. This E-service was developed to provide 24-hour counseling, education, and prevention, as well as to alleviate anxiety, resolve ambiguities, and dispel rumors by providing accurate and scientific responses to people’s questions [58].

b) *Diagnosis and Detection*

Various consultation systems have been developed to assist in diagnosing infections or suspected infections [13, 18, 21, 59]. For instance, the “4030” call service is an appropriate digital solution for providing information on the initial diagnosis of COVID-19 via individual symptoms, thereby assisting the general public and individuals with special needs, such as pregnant women and older adults [21]. “Mashverapp” is a web-based medical and psychological counseling service that provides consultation services to patients with COVID-19. Additionally, this application provides a service for performing diagnostic tests at home [18]. “Corona’s online testing program” was developed in collaboration with professors and the medical system organization. It is based on the most recent diagnostic algorithm. By asking a few medical questions and determining whether a patient requires a face-to-face visit with a physician, this program aims to reach a clinical decision [19, 20].

iii) *Treatment*

During the COVID-19 pandemic, E-health solutions based on consultation services were used to accomplish treatment objectives such as the guide to nutritional issues and the treatment of other chronic disorders [58]. As a result, the State Welfare Organization (SWO) developed an intelligent electronic system for Iranians to self-assess their psychological well-being during the COVID-19 outbreak. The purpose of this system is to develop appropriate content regarding an individual’s mental health status, to conduct appropriate screenings based on scientific evidence, to provide expert and scientific psychological advice, and to prevent psychosocial disorders associated with the COVID-19 pandemic [24]. The National Headquarters for Coronavirus Controls’ 42nd session resolution addressed the issue of “electronic care of quarantined individual”. As a result, the Ministry of Communications and Information Technology (MOCIT) is required to provide the Ministry of Health and Medical Education (MOHME) with multiple electronic monitoring services for mobility management during quarantine periods [59].

iv) *Management and planning*

The MOHME has established a system for COVID vaccination registration. Iranians can use this system to register their national identification code, date of birth, and mobile phone number. Afterward, subscribers will be notified via SMS of the reserved vaccine time and location [22, 60].

Moreover, an individual’s quarantine status can be determined through the “Mask” application. “The National Headquarters for Coronavirus Control” is an organization formed in early February 2020 in response to an outbreak of coronavirus in Iran. With the approval of the Supreme National Security Council, the organization has mandated that all laboratories register the National Patient Code in the integrated system of the Ministry of Health and the Ministry of Communications [59].

Additionally, the economic status of patients can be determined by combining information about infected cases announced by the Ministry of Health with data from the Iranian Welfare Database [59]. The Ministry of Communications used dashboards, surveillance systems, and the capability of telecommunication tools and mobile networks to visualize the outbreak map, identify provinces with the most incoming and outgoing passengers, determine the country’s busiest locations, and calculate outbreak risk points [59].

### Phase 3. The proposed framework for IT-based management of COVID-19

**Fig. 3:**
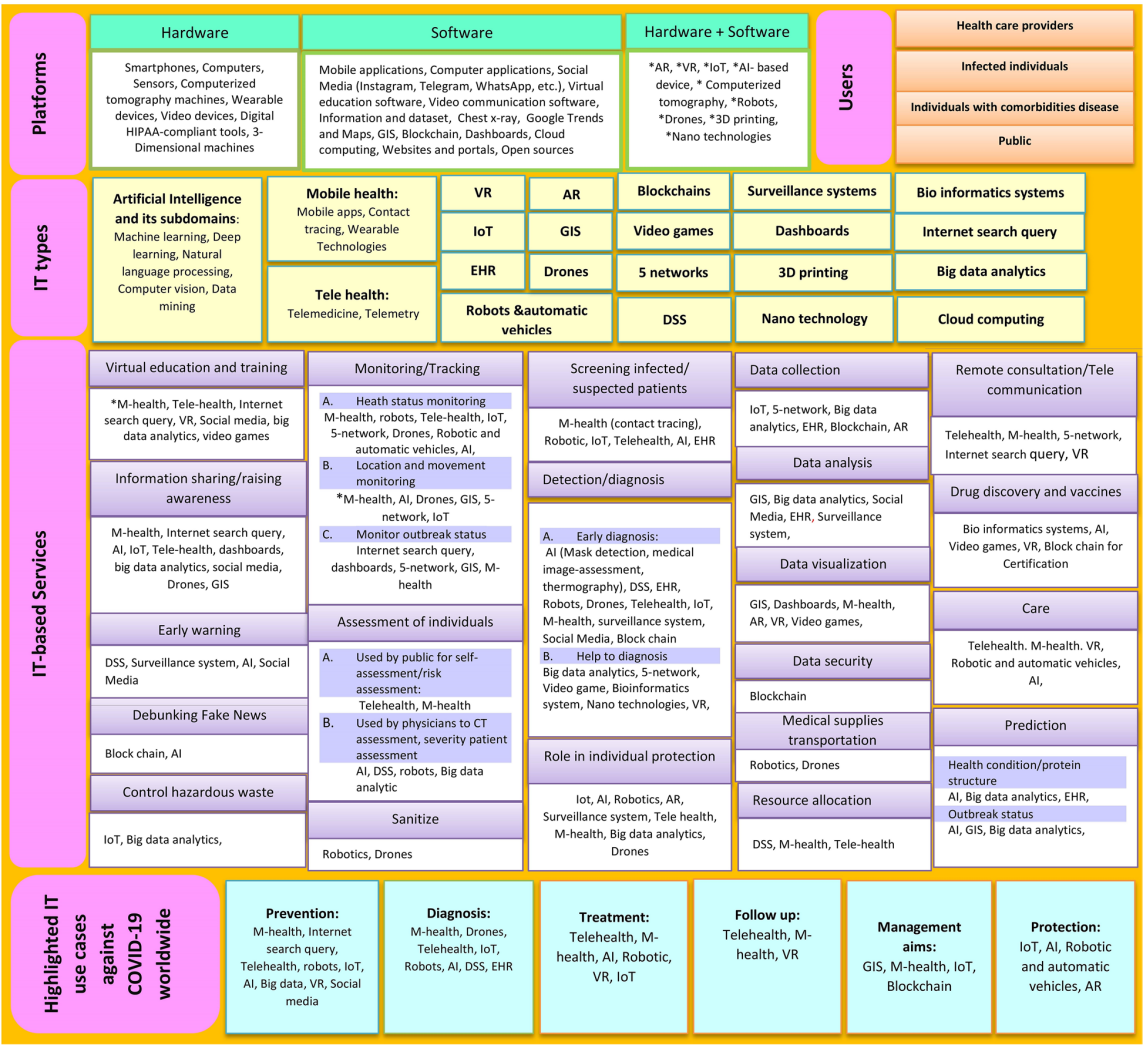
Proposed Conceptual Framework based on practical experiences of IT for the management of the COVID-19 epidemic

As illustrated in Fig. 3, a comprehensive framework for managing the COVID-19 was proposed based on extensive literature review and expert knowledge. As the framework demonstrates, 22 technologies (i.e., AI and its subdomains, M-health, Telehealth, VR, AR, IoT, EHR, GIS, robots, drones, blockchains, video games, five networks, DSS, surveillance system, dashboards, 3D printing, nanotechnology, bioinformatics system, Internet search query, big data analytics, and cloud computing) can be used to combat the COVID-19 outbreak. By providing a variety of services, such as virtual education and training, information sharing/awareness-raising, early warning, debunking fake news, controlling the hazardous waste, monitoring/tracking, individual assessment, sanitizing, screening infected/suspected individuals, detection/diagnosis, individual protection, data collection, data analysis, data visualization, data security, transport medical supplies, resource allocation, and remote consultation, these technologies assist in the management of the COVID-19 outbreak (see Table 1 for details).

Six categories of variables were identified: prevention, diagnosis, treatment, follow-up, management and planning, and protection. Specialized technologies are highlighted for each group. For example, prevention objectives have emphasized mobile health, Internet search queries, telehealth, robots, IoT, AI, big data, VR, and social media (see Table 1; Fig. 3 for details). Finally, the experts confirmed the proposed framework (*n *= 13), and their comments were adapted.

## Discussion

This study conducts a comprehensive literature review to demonstrate the capabilities of IT in managing the COVID-19 pandemic. A conceptual framework for an IT-based pandemic response is proposed based on a literature review and expert knowledge (see Figs. 2 and 3). Iran’s practical experience shows that various types and capabilities of IT were not used to respond to the COVID-19 pandemic. IT applications used against the COVID-19 outbreak are classified into six core topics in our proposed framework, namely: (1) Prevention, (2) Diagnosis, (3) Treatment, (4) Follow-up, (5) Management, and (6) Protection.

Technologies highlighted for prevention purposes include M-health [4, 9, 16–28], Internet search query, Telehealth [9, 13, 17, 20, 22, 29–33], robots [9, 13, 16, 23, 29, 30], IoT [9, 23, 29, 30, 34–36], AI [16, 23, 29, 30, 35, 37–43], big data analytics [4, 29, 30, 35, 42, 44], and social media [13, 17, 21, 29]. M-health, internet search queries, and social media have all been used in Iran during the current pandemic. According to a study, VR, robotics, infection control systems, and AI methods should be considered more heavily during prevention [9]. Additionally, their findings indicated that prior to the COVID-19 pandemic, there were no pre-crisis efforts directed at COVID-19 prevention. Strategic planning is required to maximize the capabilities of Iran’s IT n order to contain the outbreak’s spread.

Various technologies have been considered to aid in the diagnosis of COVID-19, the most prominent of which are M-health [9, 16–22, 24, 27, 28, 31, 47], Telehealth [9, 17, 20, 22, 30, 31, 33], AI [23, 29, 30, 37–40, 42, 43, 48–50], drones [29], IoT [4, 9, 23, 30, 34, 36], and robots [9, 16, 29]. According to a study, AI, web-based genome detectives, Telehealth, M-health, IoT, surveillance systems, robotics, and DSS were all used to diagnose and detect COVID-19 patients until July 2020 [9]. Ye et al. (2020) reported that AI-based scenarios, such as drones, intelligent diagnosis (e.g., deep learning-based computer-aided diagnostic system), temperature detection (e.g., airport infrared thermal cameras), and robots (e.g., decontamination, medication delivery, and vital sign assessment), were critical in the detection and diagnosis of COVID-19 in China [14]. In Iran, E-Health is used to diagnose infected individuals or those suspected of being infected, while a chest CT scan is used to diagnose COVID-9. However, many potentials of IT have not been utilized for diagnostic purposes in this country; consequently, additional opportunities for using IT for diagnostic purposes are required in this country. For instance, AI should be used to determine the severity of COVID-19 (see Fig. 3).

By providing various services, IT has aided in treating COVID-19 disease. We understand that effective treatment is contingent upon the discovery of COVID inhibitors. As a result, the use of information technologies such as AI [29, 37, 40], and VR [45] have a facilitating role in vaccine discovery. Other technologies, including M-health [9, 16–24, 27, 31], and telemedicine [9, 13, 20, 22, 30–33], are also effective approaches in treating patients at home. Additionally, big data analytics [44], 5G networks [23], and video games [13] can be used to advance treatment goals by facilitating healthcare decision-making, teleconsultation, and drug discovery. In Iran, only a few E-health capabilities (e.g., electronic care for people in quarantine, performing health inquiry services, responding to patients via online and off-line consultation systems, and Telemental) have been used to accomplish treatment objectives during this period. Other technologies should be used to treat and monitor patients in this country (see Fig. 3).

During the COVID-19 pandemic, IT applications for management and planning were highlighted. Various technologies, such as GIS [4, 9, 17, 51, 52], M-health [9, 17–20, 23, 24, 27, 28], blockchain [4, 23, 30, 35, 46], and IoT [4, 9, 29, 30, 34–36] have been used for a variety of purposes, including control, monitoring, and tracking, information sharing, data visualization, and enabling a secure donation platform. Moreover, the use of DSS [9, 35], big data analytics [4, 29, 30, 35, 44], surveillance system [9], 5G network [23], Internet search query [9], video games [13], and EHR [53] are examples of additional digital solutions that can aid in decision making, data analysis, prediction, and resource allocation. Management and control of pandemic status are impossible without using IT capabilities. In other words, information technology can aid governments and decision-makers in decision-making and pandemic management by providing real-time data, facilitating information sharing, data analysis, and knowledge about valuable tools for pandemic control.

In Iran, the benefits of IT for managing and controlling the COVID-19 outbreak have been prioritized over other objectives. Consequently, decision-makers may benefit from using databases, dashboards, surveillance systems, E-health, and HIS to manage the COVID-19 disease. It is worth noting that one of Iran’s primary challenges is the proliferation of databases and information resources, which has resulted in a dearth of accurate information. As a result, it is necessary to integrate this data using data mining techniques and AI to create valid databases. Furthermore, the use of IT to control the costs of consumables and medications should be considered. Moreover, centralized and online control of hospital beds is required to increase hospital productivity. Subsequently, our proposed framework calls for the adoption of additional digital solutions in this country.

IT has also aided in the achievement of protection goals; for example, the use of drones [23] and robots [9, 16, 23, 29] reduced the workload of healthcare workers and minimized human interaction; similarly, other technologies such as AI [23, 29, 35, 37–40, 42], and IoT [4, 9, 23, 34–36] has the potential to significantly reduce healthcare workers’ workloads and facilitate the implementation of public health interventions. Additionally, by utilizing AR, patients’ data can be sent directly to the healthcare system without any contact or paperwork in triage. Moreover, E-health [9, 20, 22, 31, 33] [17, 18, 24, 25, 27, 28] is another digital solution that can help minimize human interaction, particularly during the quarantine period. In Iran, the absence of protective measures is more pronounced. As a result, more attention should be paid to the use of assistive technologies to protect individuals against COVID-19 disease.

## Conclusions

IT has a critical role to play in the COVID-19 outbreak. In Iran, information technology has focused on four primary categories: (1) prevention through the use of some M-health capabilities, internet search queries, and social media; (2) initial diagnosis through the use of E-health capabilities and diagnostic medical imaging equipment; (3) treatment through the use of E-health capabilities; and (4) management objectives through the use of databases, E-health, dashboards, surveillance systems, and HIS use cases. According to our proposed framework, multiple digital solutions (i.e., AI and its subdomains, M-health, Tele-health, VR, AR, IoT, EHR, Robots, drones, Blockchains, and video games, among others) should be used in a variety of fields, including prevention and detection, diagnosis, treatment, follow-up, and management, as well as the protection objectives during the COVID-19 outbreak. It should be noted that IT applications are an unavoidable component of COVID-19 management.

To summarize, various IT applications such as AI, IoT, VR, AR, DSS, blockchain, robots, drones, and video games have been ignored in Iran, as has the full potential of utilized technologies, including E-health and surveillance systems. As a result, to respond to this pandemic, Iran’s government and decision-makers should consider strategic planning that incorporates successful experiences against COVID-19 and the most advanced IT capabilities.

## Data Availability

The data that supported the findings of this study are available from the corresponding author on request.
